# Caffeic acid phenethyl ester promotes oxaliplatin sensitization in colon cancer by inhibiting autophagy

**DOI:** 10.1038/s41598-024-65409-2

**Published:** 2024-06-25

**Authors:** Fei Xing, Ning Liu, Can Wang, Xu-Dong Wang

**Affiliations:** 1 Department of Gastrointestinal Nutrition Surgery, The Second Hospital of Jilin University, Changchun, 130000 China; 2 Academic Center, The Second Hospital of Jilin University, Changchun, 130000 China

**Keywords:** Cancer, Gastroenterology

## Abstract

Colon cancer ranks as the third most prevalent form of cancer globally, with chemotherapy remaining the primary treatment modality. To mitigate drug resistance and minimize adverse effects associated with chemotherapy, selection of appropriate adjuvants assumes paramount importance. Caffeic acid phenethyl ester (CAPE), a naturally occurring compound derived from propolis, exhibits a diverse array of biological activities. We observed that the addition of CAPE significantly augmented the drug sensitivity of colon cancer cells to oxaliplatin. In SW480 and HCT116 cells, oxaliplatin combined with 10 µM CAPE reduced the IC_50_ of oxaliplatin from 14.24 ± 1.03 and 84.16 ± 3.02 µM to 2.11 ± 0.15 and 3.92 ± 0.17 µM, respectively. We then used proteomics to detect differentially expressed proteins in CAPE-treated SW480 cells and found that the main proteins showing changes in expression after CAPE treatment were p62 (SQSTM1) and LC3B (MAP1LC3B). Gene ontology analysis revealed that CAPE exerted antitumor and chemotherapy-sensitization effects through the autophagy pathway. We subsequently verified the differentially expressed proteins using immunoblotting. Simultaneously, the autophagy inhibitor bafilomycin A1 and the mCherry-EGFP-LC3 reporter gene were used as controls to detect the effect of CAPE on autophagy levels. Collectively, the results indicate that CAPE may exert antitumor and chemotherapy-sensitizing effects by inhibiting autophagy, offering novel insights for the development of potential chemosensitizing agents.

## Introduction

According to the latest report of the International Cancer Statistics 2022, the total number of new colon cancer cases is 1.9 million and ranks as the third most prevalent type of cancer^1^. Most colon cancers are detected in the middle and late stages, which poses a great challenge for treatment. Surgical intervention is a viable option for early-stage colon cancer, while advanced cases with distant metastasis necessitate systemic therapy such as chemotherapy, targeted therapy and immunotherapy^2–4^. Commonly used chemotherapeutic agents include oxaliplatin, 5-fluorouracil, irinotecan, and docetaxel^5^. With the increase in the use of targeted drugs and immunotherapy, more choices are available for the comprehensive treatment of colon cancer^6^. However, platinum-based chemotherapy continues to be the preferred initial treatment option for drug therapy in colon cancer^7^. The resistance to platinum-based chemotherapeutic agents, as well as the toxic side effects of these agents, have emerged as important factors impacting patient prognosis and treatment adherence^8^. Therefore, selecting appropriate chemotherapeutic sensitizers is particularly important.

The pharmacological effects of natural products are diverse, and many of them are highly promising for the treatment of chronic diseases^9–11^. Metabolites of various natural products exhibit antitumor effects in cancers^12–15^. Caffeic acid phenethyl ester (CAPE), one of the main active ingredients of propolis, was originally considered to be an inhibitor of NF-κB, and has since been discovered to possess antiviral, antibacterial, anticancer, and immunomodulatory properties^16–19^. Numerous studies have demonstrated the potential of CAPE as a chemosensitizer in conjunction with chemotherapy agents^20–22^. The sensitivity of gastric cancer cells to adriamycin (DXR) and cisplatin (CDDP) can be enhanced by CAPE through the reduction of proteasome activity^23^. The combination of CAPE with vincristine and paclitaxel exhibits a synergistic effect in the treatment of prostate cancer^24^. The down-regulation of claudin-2 by CAPE can enhance the sensitivity of lung cancer cells to DXR^25^. Additionally, CAPE causes breast cancer cells to be more responsive to docetaxel^26^. Studies have demonstrated that CAPE exerts anti-tumor effects in colon cancer cells through the regulation of AMPK activity and the β-catenin pathway^27,28^. However, its potential for enhancing chemosensitivity in colon cancer remains unexplored. In this study, we discovered that CAPE can augment the sensitivity of colon cancer cells to oxaliplatin both in vivo and in vitro. Additionally, we employed proteomics to screen for potential targets of CAPE, providing valuable insights for its clinical application in colon cancer (Supplementary Information).

## Results

### CAPE increases oxaliplatin chemo-sensitivity in colon cancer in vitro

**Figure 1:**
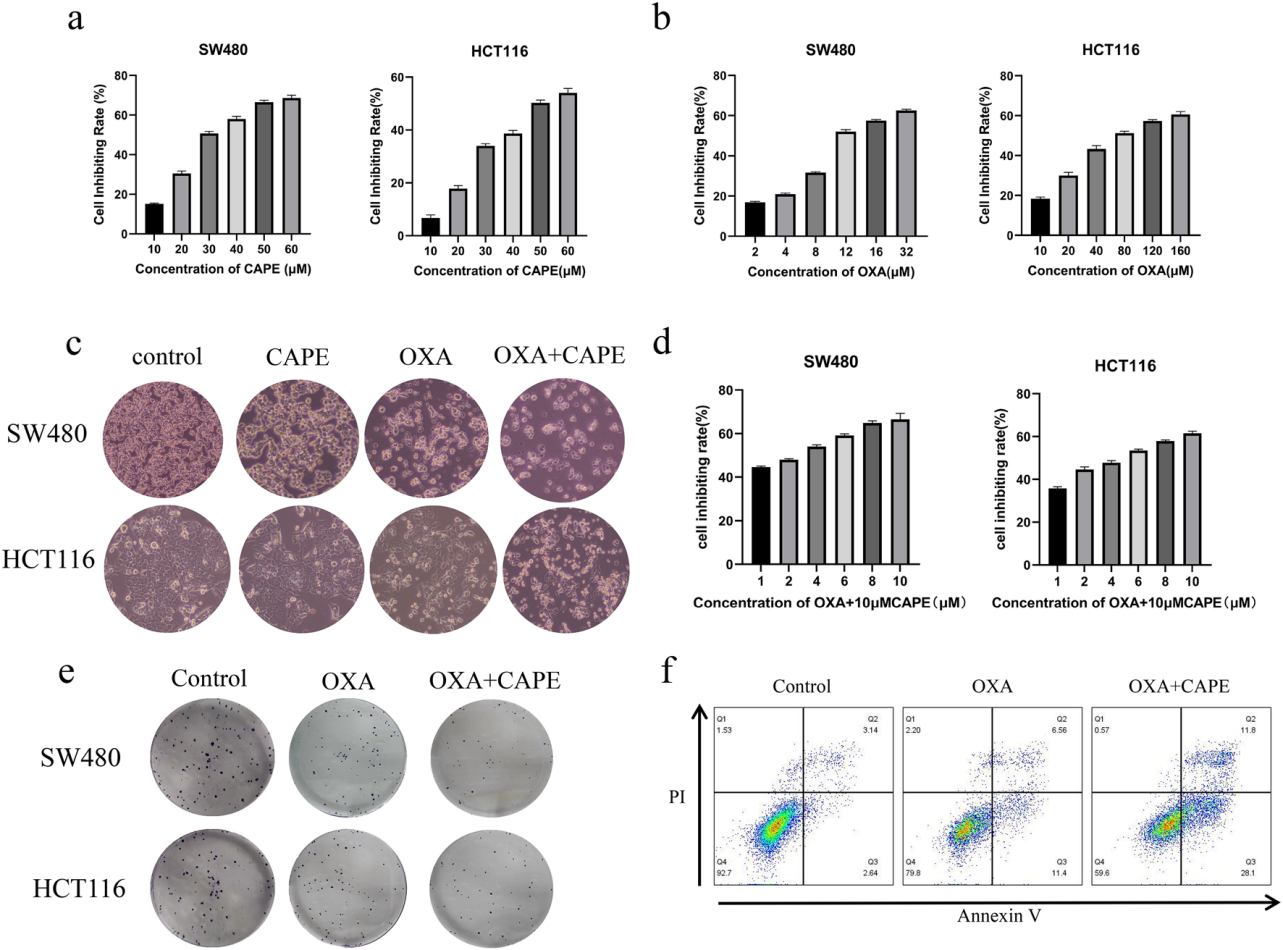
CAPE increases OXA chemo-sensitivity in colon cancer in vitro. (**a**) The inhibitory rate of CAPE exposure for 24 h on SW480 and HCT116 cells. (**b**) The inhibitory rate of OXA exposure for 24 h on SW480 and HCT116 cells. (**c**) The morphological changes of SW480 and HCT116 cells treated with OXA, CAPE, and OXA + CAPE for 24 h were observed using a microscope. In SW480 cells, the concentrations used were 2 µM OXA, 10 µM CAPE, and a combination of 2 µM OXA + 10 µM CAPE. Similarly, in HCT116 cells, the concentrations used were 4 µM OXA, 10 µM CAPE, and a combination of 4 µM OXA + 10 µM CAPE. (**d**) Cytostatic rate of 24 h exposure to OXA combined with 10 µM CAPE in SW480 and HCT116 cells. (**e**) A colony formation assay was performed on SW480 and HCT116 cells treated with OXA, CAPE, and OXA + CAPE. In SW480 cells, the concentrations used were 2 µM of OXA, 10 µM of CAPE, and a combination of 2 µM of OXA + 10 µM of CAPE. Similarly, in HCT116 cells, the concentrations used were 4 µM of OXA, 10 µM of CAPE, and a combination of 4 µM of OXA + 10 µM of CAPE. (**f**) Detection of apoptosis in SW480 cells after treatment with 2 µM OXA or 2 µM OXA combined with 10 µM CAPE for 24 h. *CAPE* caffeic acid phenethyl ester, *OXA* oxaliplatin.

Initially, we observed that CAPE inhibited the growth of colon cancer in a dose-dependent manner. The IC_50_ values of CAPE treatment for SW480 and HCT116 cells after 24 h were determined to be 32.26 ± 0.63 µM and 51.74 ± 1.19 µM (Fig. 1a), respectively. Similarly, the IC_50_ values of SW480 and HCT116 cells treated with oxaliplatin (OXA) for 24 h were found to be 14.24 ± 1.03 µM and 84.16 ± 3.02 µM (Fig. 1b), respectively. After treatment with 10 µM CAPE, no significant tumor inhibition was observed in SW480 and HCT116 cells (Fig. 1c). Therefore, we chose 10 µM CAPE as the co-administration concentration. The CCK-8 assay showed that, in SW480 and HCT116 colon cancer cells, OXA combined with 10 µM CAPE reduced the IC_50_ of OXA from 14.24 ± 1.03 and 84.16 ± 3.02 µM to 2.11 ± 0.15 and 3.92 ± 0.17 µM, respectively (Fig. 1d). The combination of OXA and CAPE enhanced apoptosis induction in colon cancer cells compared with treatment with OXA alone, as observed using light microscopy (Fig. 1c). The clone formation assay confirmed that CAPE combined with OXA treatment significantly increased the colony growth-inhibitory effect of OXA on the SW480 and HCT116 cell lines (Fig. 1e). We then used flow cytometry to explore the sensitizing effect of CAPE on OXA. The combination of CAPE and OXA enhanced apoptosis in SW480 colon cancer cells as compared with OXA alone (Fig. 1f). Overall, these findings suggested that CAPE increased OXA chemo-sensitivity in colon cancer in vitro*.*

### CAPE increases oxaliplatin chemo-sensitivity in colon cancer in vivo

**Figure 2:**
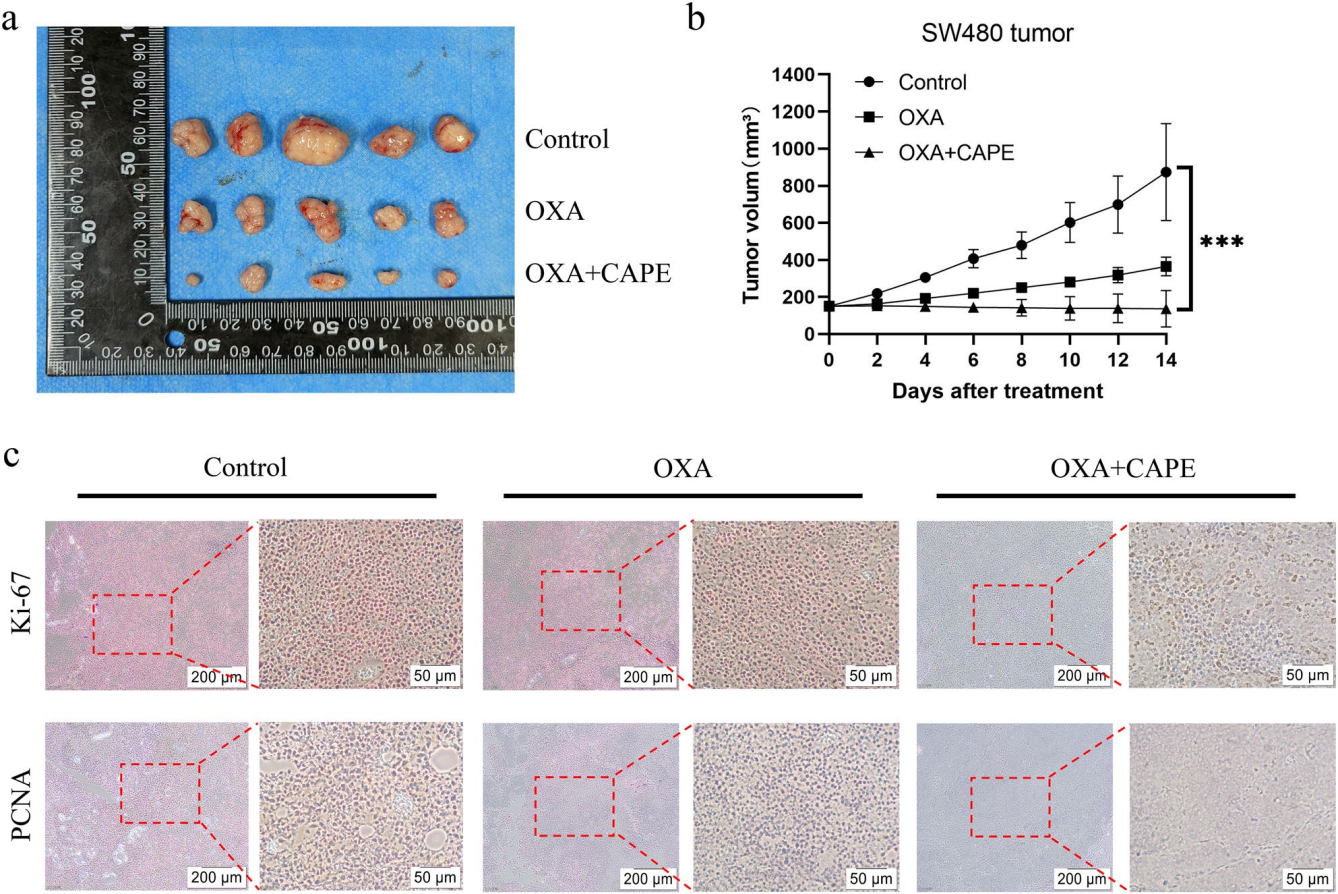
CAPE increases OXA chemo-sensitivity in colon cancer in vivo. (**a**) SW480 cells were used to construct xenograft tumor mouse models and were administered according to experimental instructions. (**b**) Tumor volume in mice with xenograft tumors. (**c**) Immunohistochemical detection of Ki67 and PCNA in xenograft tumors. *CAPE* caffeic acid phenethyl ester, *OXA* oxaliplatin.

The xenograft tumor mouse model was established by utilizing the SW480 cells. Mice were randomly assigned to three groups (n = 5 per group): control, OXA (10 mg/kg), or OXA (10 mg/kg) + CAPE (10 mg/kg) when the tumor volume reached approximately 150 mm^3^. The volume of transplanted tumors was measured 14 days post drug treatment. Compared to the control group (873.6 ± 261.6 mm^3^) and OXA group (364.6 ± 44.9 mm^3^), the tumor growth in the OXA + CAPE group (136.2 ± 98.1 mm^3^) exhibited significant inhibition(Fig. 2a,b), with a statistically significant difference observed (P < 0.001).

In addition, immunohistochemistry of mouse transplanted tumors showed that the levels of the proliferation markers Ki67 and PCNA in the combined treatment group were significantly reduced as compared with those in the control and OXA monotherapy groups (Fig. 2c). These findings suggest that CAPE increases drug sensitivity to OXA in vivo, which has potential clinical therapeutic implications for patients with colon cancer.

### Identification of CAPE targets in colon cancer using proteomics

**Figure 3:**
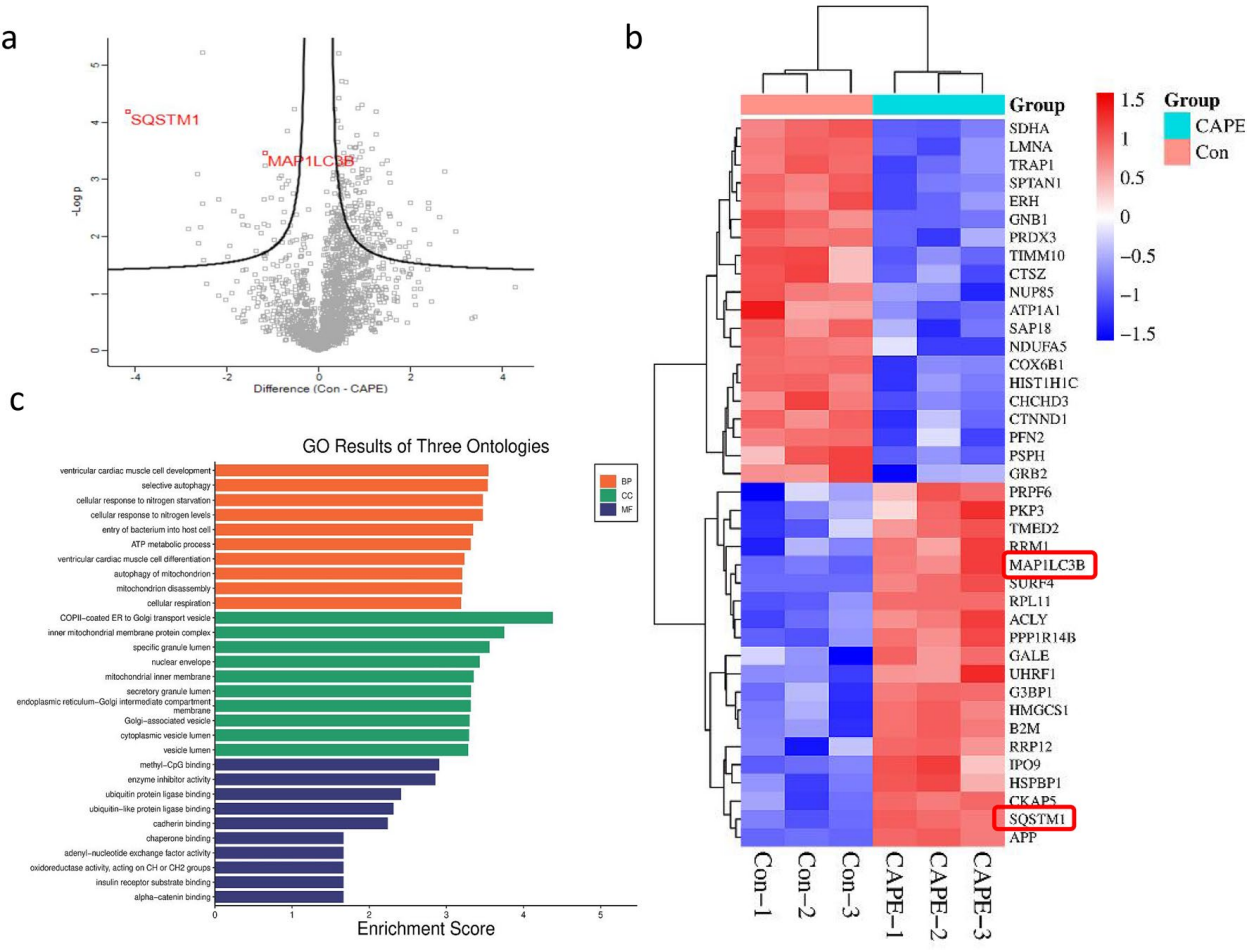
Mass spectrometry analysis of CAPE-treated target proteins. (**a**) Volcano plot of differential proteins following a 24 h treatment of SW480 cells with 10 µM CAPE. (**b**) Heat map of the 40 differentially expressed proteins that changed the most following a 24 h treatment of SW480 cells with 10 µM CAPE. (**c**) Gene Ontology analysis of 40 differentially expressed proteins of which the expression changed the most after CAPE treatment. *CAPE* caffeic acid phenethyl ester. [The (**b**) was generated with http://www.bioinformatics.com.cn/].

We next employed proteomics to identify differentially expressed proteins following CAPE treatment. Previously, using the CCK-8 assay, we had determined that a concentration of 10 µM CAPE combined with OXA for 24 h demonstrated notable effects on SW480 cell proliferation. Therefore, this concentration was selected as the optimal drug dose for the CAPE treatment group. After treatment with 10 µM CAPE for 24 h, protein extraction was performed and MS pretreatment and analysis were conducted according to the enzyme digestion method. A total of 1852 proteins were identified using proteomic analysis. Compared with the control group, 34 upregulated proteins and 166 downregulated proteins were observed in the CAPE-treated group (Fig. 3a). Differential proteins are listed in Supplementary Table 1. Notably, p62 and LC3B levels showed significant up-regulation after CAPE treatment (Fig. 3b). We further subjected the top-40 significantly upregulated and downregulated proteins to Gene Ontology (GO) enrichment analysis, which revealed that selective autophagy, ATP metabolic process, cellular response to nitrogen starvation and other pathways were primarily involved in mediating the antitumor effects exerted by CAPE (Fig. 3c).

### Validation of differentially expressed proteins

**Figure 4:**
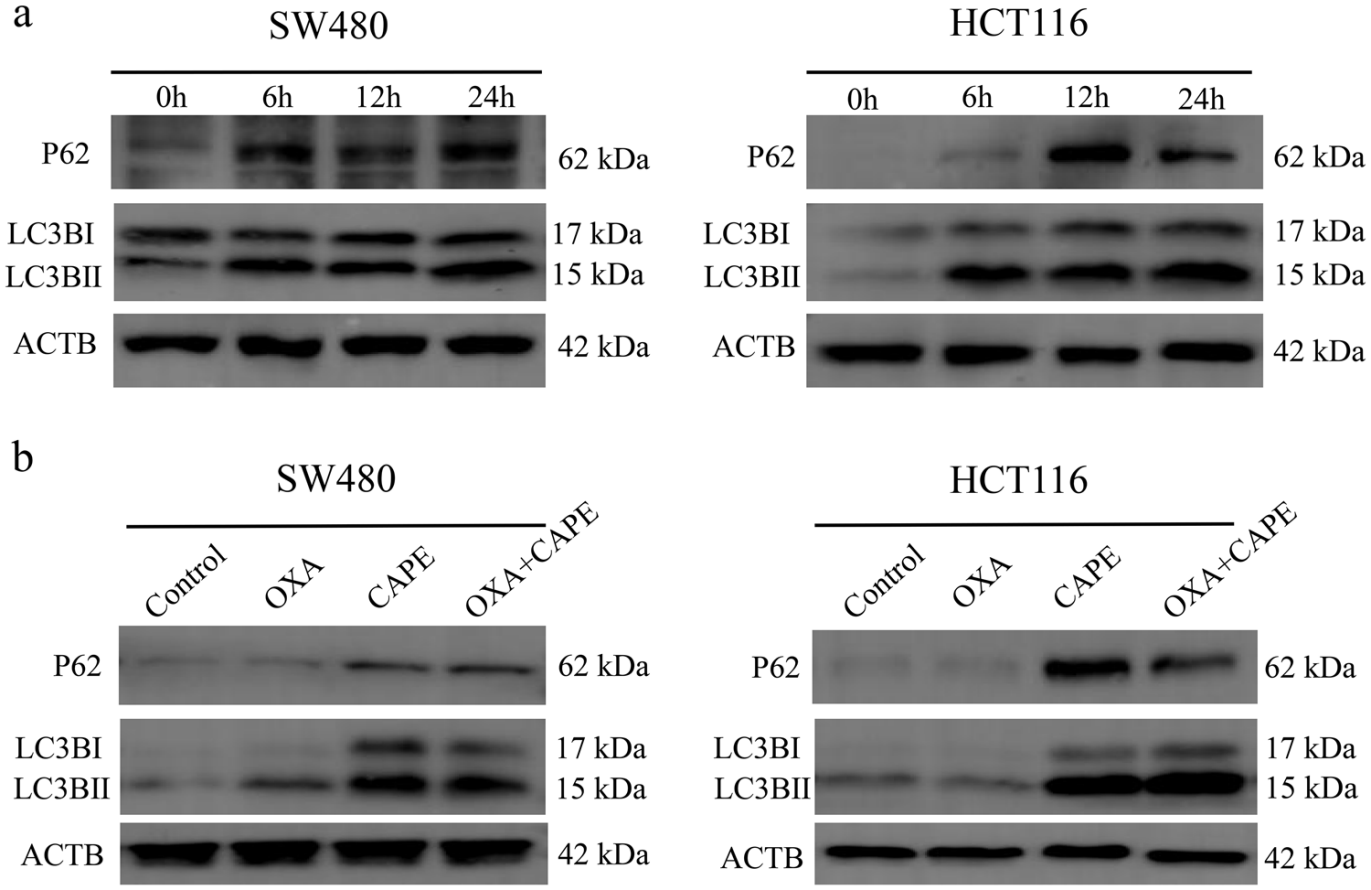
CAPE treatment upregulated p62 and LC3B expression in colon cancer. (**a**) Immunoblotting of p62 and LC3B in SW480 and HCT116 cells treated with 10 µM CAPE for 0, 6, 12, and 24 h. (**b**) Immunoblotting of p62 and LC3B in SW480 and HCT116 cells treated with OXA, CAPE, and OXA + CAPE for 24 h. In SW480 cells, the concentrations used were OXA (2 µM), CAPE (10 µM), and OXA (2 µM) + CAPE (10 µM). Similarly, in HCT116 cells, the concentrations used were OXA (4 µM), CAPE (10 µM), and OXA (4 µM) + CAPE (10 µM). *CAPE* caffeic acid phenethyl ester, *OXA* oxaliplatin.

Using proteomics, we determined that CAPE may exert chemosensitizing effects by targeting p62 and LC3B. We then verified the proteomic results by using immunoblotting. In SW480 and HCT116 cells, both p62 and LC3B-II were upregulated after CAPE treatment for 6 h (Fig. 4a). Compared with those in the control or OXA groups, p62 and LC3B-II proteins in the CAPE group and the OXA + CAPE combined group were significantly upregulated after 24 h of drug treatment (Fig. 4b). This indicated that CAPE exerted a chemosensitizing effect by regulating p62 and LC3B protein levels.

### CAPE suppresses late autophagy in colon cancer cells

**Figure 5:**
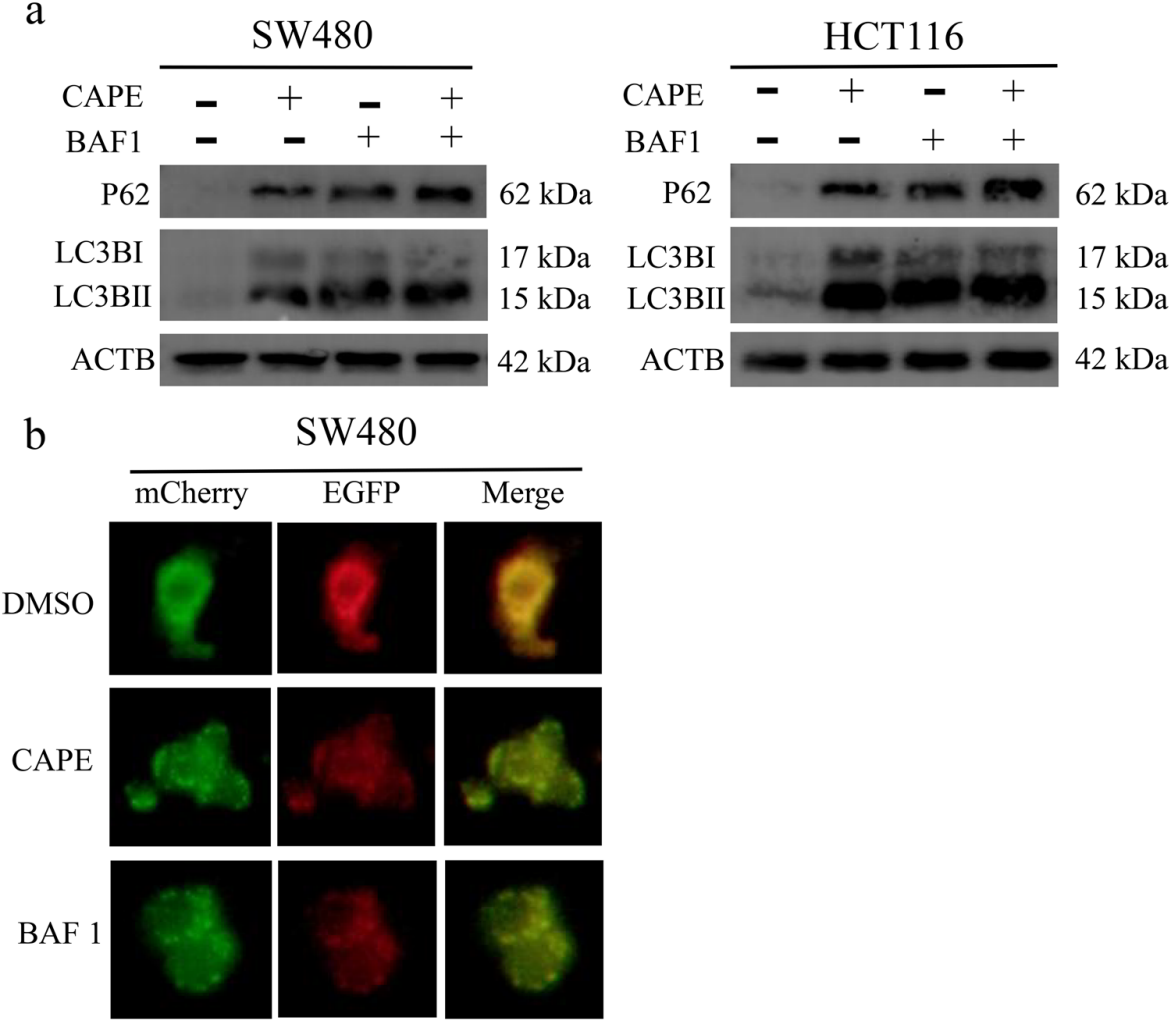
CAPE suppresses autophagy in colon cancer cells. (**a**) Changes in p62 and LC3B protein levels after treatment of colon cancer cells with CAPE (10 μM) or BAF 1 (10 nM) for 24 h. (**b**) SW480 cells overexpressing mCherry-EGFP-LC3 were treated with CAPE (10 μM) or BAF 1 (10 nM) for 24 h and then examined by fluorescence microscopy. *CAPE* caffeic acid phenethyl ester, *BAF 1* bafilomycin A1.

Through proteomic and GO analyses, we found that the upregulated proteins p62 and LC3B in the autophagy pathway were enriched after CAPE treatment, indicating that CAPE may exert its drug-sensitizing effects by regulating autophagy in colon cancer cells. We introduced the autophagy inhibitor bafilomycin A1 (BAF1) as a control and found that, after treating SW480 and HCT116 colon cancer cells with 10 µM CAPE or 10 nM BAF1 for 24 h, the levels of both p62 and LC3B proteins were significantly upregulated (Fig. 5a).

**Figure 6:**
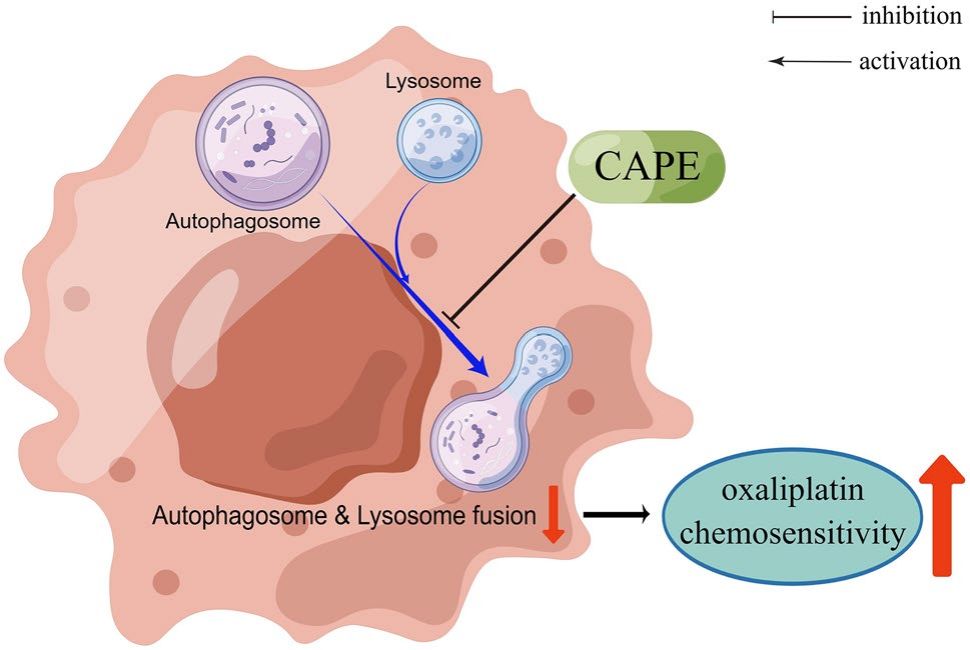
Mechanism by which CAPE inhibits autophagy and promotes OXA sensitivity in colon cancer cells. The figure was drawn by Figdraw. *CAPE* caffeic acid phenethyl ester, *OXA* oxaliplatin.

SW480 cells transfected with the mCherry-EGFP-LC3B dual-luciferase system also demonstrated that, following 24 h of CAPE or BAF1 treatment, a significant decrease in red fluorescence, an increase in green fluorescence, and a notable augmentation of intracellular autophagosomes were observed (Fig. 5b). This indicates that CAPE plays a role similar to the late autophagy inhibitor BAF 1, thereby inhibiting the combination of autophagosomes and lysosomes, inhibiting the autophagy flux of colon cancer cells, and improving chemotherapy sensitivity (Fig. 6).

## Discussion

Currently, platinum-based chemotherapy regimens are still used to treat patients with intermediate and advanced colon cancer. Side effects caused by platinum drugs pose challenges to first-line clinical treatments^29–31^. Compared with traditional chemotherapy drugs, natural products have the characteristics of presenting a wide range of synthetic raw materials, fewer side effects, and diverse therapeutic targets^32,33^. Selecting appropriate natural products as chemotherapy adjuvants can greatly reduce the side effects of chemotherapeutic drugs and improve their antitumor effects. As the main active ingredient of propolis, CAPE targets multiple signaling pathways to inhibit cancer progression^34,35^. Currently, the underlying mechanisms of CAPE in colon cancer remain unclear. Through a CCK-8 experiment, we found that CAPE has antitumor and OXA-sensitizing effects on colon cancer cells, which aroused our research interest. We here confirmed that CAPE can enhance the chemo-sensitivity of colon cancer to OXA and promote apoptosis in tumor cells. These findings have important clinical implications.

Among the MS results, two proteins, p62 and LC3B, exhibited the most significant alterations after CAPE treatment compared with those in the control group, which was notable. Western blot analysis revealed significant upregulation of p62 and LC3B in both the CAPE group and OXA + CAPE group after 24 h of treatment. Therefore, we propose that CAPE exerts its chemosensitizing effect by modulating the expression of p62 and LC3B.

p62 and LC3B serve as autophagy markers. The p62 protein contains multiple domains and acts as an adaptor between the autophagosome and substrate, playing a crucial role in regulating autophagy^36–38^. However, LC3B-I undergoes lipidation, similar to ubiquitination, resulting in the formation of lipidated LC3B-II that attaches to membranes and functions as a structural protein within autophagosomes. Following CAPE treatment, both p62 and LC3B were significantly upregulated, indicating normal initiation of autophagy, but impaired fusion between autophagosomes and lysosomes, suggesting the inhibition of autophagosomal degradation. By employing the mCherry-EGFP-LC3 reporter gene, we observed decreased red fluorescence intensity, increased green fluorescence intensity, and an elevated number of punctate autophagosomes after CAPE treatment. These findings suggested that CAPE treatment hinders cellular autophagic flux. BAF 1 is an inhibitor of late stages of autophagy that blocks the fusion of autophagosomes with lysosomes^39^. By comparison with BAF 1, we demonstrated that CAPE treatment also impeded late-stage autophagy in colon cancer cells.

The degradation of biological macromolecules and organelles is the main function of autophagy in eukaryotes^40^. Tumor cells modulate cell proliferation and apoptosis by regulating autophagy under various conditions^41^. Autophagy induction is also the reason for drug resistance in tumor cells, and autophagy inhibitors have been confirmed to have chemotherapy-sensitizing effects on a variety of tumor cells^42,43^. This suggests that autophagy inhibition may be an important means of treating tumors. Autophagy-related proteins and drugs have also increasingly attracted attention^44^. The circular RNA circATG4B promotes autophagy in colon cancer and induces oxaliplatin resistance through the encoded circATG4B-222aa^45^. Trimethomide-I inhibits colon cancer by inhibiting lysosomal hydrolases and causing the accumulation of autophagic lysosomes, thereby synergizing with 5-fluorouridine or doxorubicin^46^. Combined treatment with autophagy inhibitors may be beneficial for patients with microsatellite-stable colon cancer who develop chemotherapy resistance^47^. In this study, we have preliminarily demonstrated that CAPE enhances the chemosensitivity of colon cancer cells to oxaliplatin by inhibiting autophagy.

Like most preclinical studies, this study has certain limitations. First, tumor cells exhibit heterogeneity, and different mechanisms of drug resistance and chemotherapy sensitization may exist among various colon cancer cell types. Therefore, our findings with SW480 cells may not necessarily apply to other colon cancer cell lines. Second, the expression of protein profiles can vary under different concentrations and durations of drug exposure. Thus, there are inherent limitations in this experiment due to the use of fixed concentration and duration for exploring the mechanism of CAPE action. In future studies on the mechanism of CAPE in colon cancer, it is necessary to investigate multiple colon cancer cells as well as varying drug concentrations and exposure times.

Our findings can be concluded that CAPE exerted a dose-dependent inhibition on the proliferation of colon cancer cells. Moreover, the combination of CAPE and OXA enhanced apoptosis in colon cancer cells, while CAPE also increased the sensitivity of colon cancer cells to OXA in vivo. Through proteomics analysis and the use of the mCherry-EGFP-LC3B dual-luciferase system, we observed that CAPE impeded the autophagy flux of colon cancer cells. These findings suggested that CAPE acted as an autophagy inhibitor, thereby enhancing the responsiveness of colon cancer cells to OXA chemotherapy. These results also represent pivotal groundwork for future inquiries into the mechanism underlying the action of CAPE in colon cancer and offer novel insights for the development of potential chemosensitizing agents.

## Methods

### Materials and reagents

CAPE (purity ≥ 99.96%, HY-N0274), OXA (purity ≥ 98%, HY17371), and BAF 1 (purity ≥ 98.92%, HY100558) were purchased from MedChemExpress (Shanghai, China). Antibodies against p62 (SQSTM1, 380612), LC3B (MAP1LC3B, 381544), and β-actin (R23613) were purchased from Zenbio (Chengdu, China). The mCherry-EGFP-LC3 lentiviral vectors were synthesized by Ubigene (Guangzhou, China).

### Cell culture

SW480 and HCT116 cells were obtained from the Chinese Type Culture Collection (Shanghai, China) All cells were cultured in DMEM medium supplemented with 10% fetal bovine serum and 1% penicillin–streptomycin (Servicebio, Wuhan, China) and incubated at 37 ℃ in a humidified chamber with 5% CO_2_^48,49^.

### Cell viability assay

The cell viability assay was prepared following other researchers with slight modifications. Briefly, cells were cultured at a density of 1 × 10^4^ cells/well in 96-well microplates (Servicebio, Wuhan, China). After drug treatment, each well received CCK-8 (10 µL; Invigentech, Irvine, CA, USA) and was incubated for 30 min at 37 ℃. A microplate reader (Bio-Rad, CA, USA) was used to measure the absorbance at 450 nm using blank wells not containing any cells. The absorbance values were used for calculating cell inhibition rates^50,51^.

### Colony formation assay

Cells were cultured at a density of 1 × 10^3^ cells/well in six-well plates for 24 h. SW480 cells were treated with CAPE (10 µM), OXA (2 µM), or OXA (2 µM) + CAPE (10 µM). HCT116 cells were treated with CAPE (10 µM), OXA (4 µM), or OXA (4 µM) + CAPE (10 µM). After a 2 week incubation period at 37 ℃ and 5% CO_2_, colonies were formed. The colonies were fixed using 4% paraformaldehyde for 20 min at 25 ℃. Staining was performed using a solution of 1% crystal violet for 30 min at 25 ℃^52^. Colonies were counted using a microscope (Leica Microsystems, Germany).

### Annexin V/PI apoptosis assay

SW480 cells were treated with oxaliplatin (2 µM) or a combination of OXA (2 µM) + CAPE (10 µM) for 24 h. The apoptosis kit from Bioss (Beijing, China) was used for staining, following the instructions provided by the manufacturer^53,54^. Flow cytometry (BD Biosciences, CA, USA) was performed to analyze the apoptotic events.

### Tumor xenograft model

Female BALB/c nude mice aged 4–6 weeks were purchased from Changchun Weishi Biotechnology Company. The in vivo mouse study was carried out in accordance with the recommendations in the Guide for the Care and Use of Laboratory Animals (8th Edition). The protocol was approved by the Ethics Committee of Changchun Weishi Technology Testing Co., Ltd. (No. 20230425-01). The study is reported in accordance with ARRIVE guidelines. Tumor xenograft models were established by the subcutaneous injection of SW480 cells (100 µL; 1 × 10^6^ cells). Mice were randomly assigned to three groups (n = 5 per group): control, OXA (10 mg/kg), or OXA (10 mg/kg) + CAPE (10 mg/kg) when the tumor volume reached approximately 150 mm^3^. Each animal was intraperitoneally injected three times a week as indicated for the control, OXA, and OXA + CAPE groups. An equivalent volume of dimethyl sulfoxide (DMSO) was administered to the control animals^55^. Tumor growth was checked every two days. After a 14 day drug treatment period, the mice were euthanized using carbon dioxide inhalation. The tumor volume was determined using the following formula: [L × (W)^2^]/2^56^.

### Immunohistochemical staining

Sections of subcutaneous tumor-bearing tissues embedded in paraffin were deparaffinized using xylene and gradually hydrated using different concentrations of alcohol. The slides were then immersed in a 3% solution of hydrogen peroxide for 10 min, followed by overnight incubation at 4 ℃ with anti-Ki67 (1:100; Zenbio) and PCNA (1:200; Zenbio). Secondary antibody was applied to the slides at 25 ℃ for 30 min. Color development was achieved by incubating with DAB solution for 3 min, followed by counterstaining with hematoxylin for 4 min^57^. Subsequently, the samples were examined under a microscope (Leica Microsystems, Germany).

### Western blotting

The proteins were separated using polyacrylamide gel electrophoresis (Epizyme, China), and subsequently transferred onto polyvinylidene difluoride membranes (Merck Millipore, USA). After blocking the membranes with 5% skim milk powder for 1 h at 37 ℃, they were incubated overnight at 4 ℃ with the primary antibodies: anti-p62 (1:1000; Zenbio) and anti-LC3B (1:1000; Zenbio). The next day, the membranes were incubated with secondary antibodies for 1 h at 37 ℃. Immunoblots were observed using an enhanced chemiluminescence kit (Epizyme)^57^.

### Pretreatment for mass spectrometry

SW480 colon cancer cells were divided into experimental and control groups. The experimental group was exposed to 10 µM CAPE for 24 h, whereas the control group received an equivalent volume of DMSO. After lysis with 8 M urea, total proteins were reduced by incubation with 5 mM tris (2-carboxyethyl) phosphine for 1 h. Subsequently, the samples were blocked using 20 mM iodoacetamide for 30 min. The samples were then hydrolyzed using porcine trypsin for 16 h at 37 ℃. The pH of the protein solution was made acidic by adding 10% trifluoroacetic acid. Peptides were desalted using ZipTips (Merck Millipore).

### Liquid chromatography with tandem mass spectrometry (LC–MS) analysis

The Orbitrap Exploris 480 mass spectrometer (Thermo Fisher, Waltham, MA, USA) was utilized to analyze isolated peptides in positive mode. The LC–MS analysis was performed at a resolution of 15,000 in data-dependent acquisition mode to examine peptide precursor ions with a charge state range of 2–6 and a minimum intensity of 8,000. MS data is available at iProX (ID = IPX0007456000).

### Autophagic flux analysis

Autophagic flux analysis was performed using the mCherry-EGFP-LC3 lentiviral expression vector (Ubigene, Guangzhou, China). SW480 cells were seeded in a 24-well plate at a density of 1 × 10^4^ cells/well and transfected with the mCherry-EGFP-LC3 lentivirus for 48 h. Hygromycin (800 µg/mL) was used to screen for stable mCherry-EGFP-LC3 transfectants. The mCherry-EGFP-LC3 positive cells were treated with CAPE (10 µM) or BAF1 (10 nM) for 24 h. Changes in autophagic flux were detected under a fluorescence microscope (Olympus, Japan)^58,59^.

### Statistical analysis

GraphPad Prism software 9.5.0 (GraphPad, CA, USA), [https://www.graphpad.com] was used for statistical analysis. Student’s t-test was used to perform statistical comparisons among different groups. The database search was conducted using MaxQuant 2.4.2.0 (Max Planck Institute for Biochemistry, Martinsried, Germany), and the resulting data from the search was processed using Perseus 2.0.10.0 software (Max Planck Institute of Biochemistry), Perseus (maxquant.org). A P-value of less than 0.05 indicated a statistically significant difference.

### Supplementary Information

Supplementary Figures.

Supplementary Table 1.

## Data Availability

The datasets are available from the corresponding author.
