# Supplementary material for: Caffeic acid phenethyl ester promotes oxaliplatin sensitization in colon cancer by inhibiting autophagy
Source: Sci Rep. 2024 Jun 25;14:14624. doi: 10.1038/s41598-024-65409-2 (PMC11199620; doi:10.1038/s41598-024-65409-2)
Supplement: Supplementary file 1 — Supplementary Figures. [file 41598_2024_65409_MOESM1_ESM.pdf]

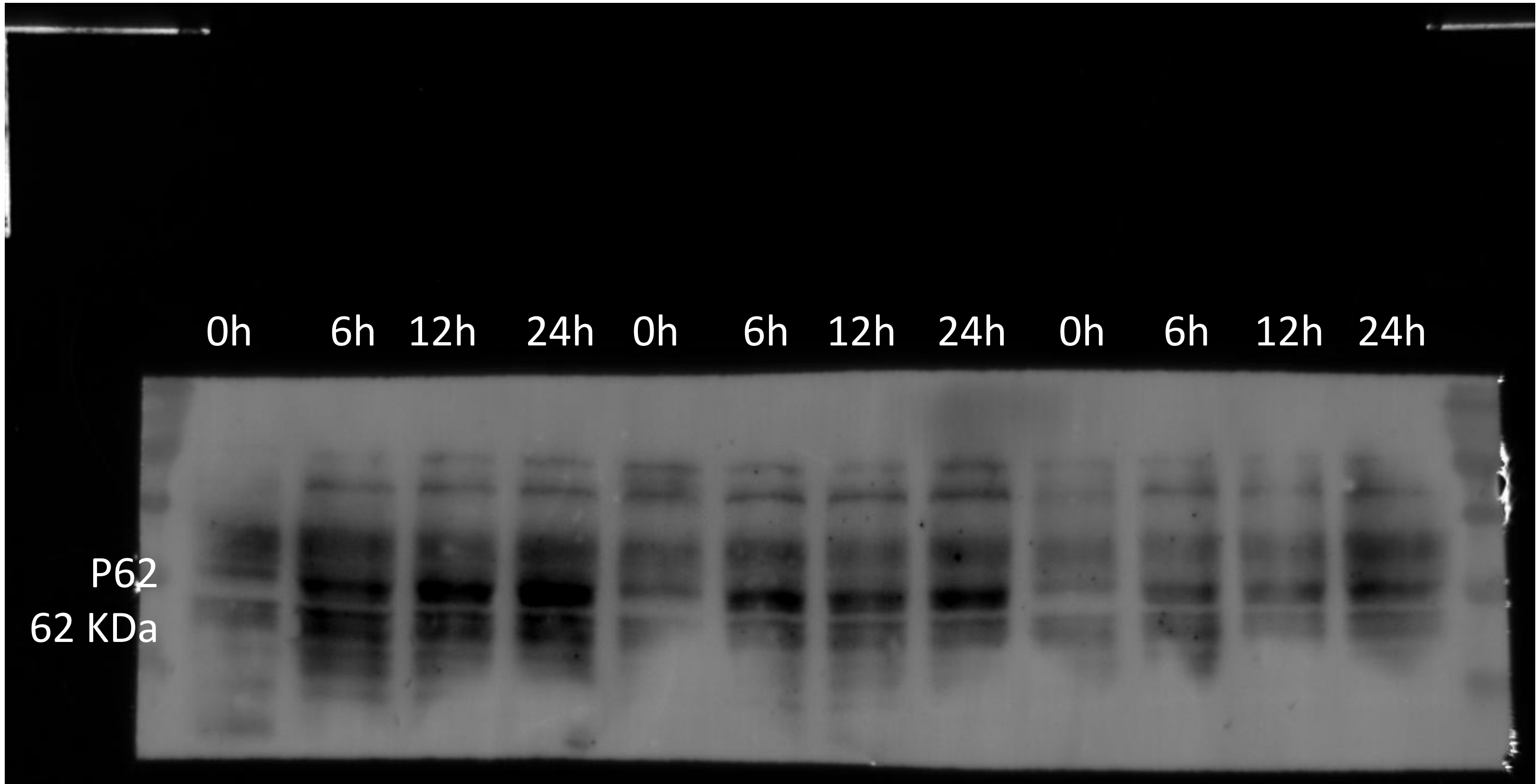

Original image of 4a. The picture is the P62 of SW480 cell line treated with CAPE at different time intervals. From left to right are 0h, 6h, 12h, 24h. Each replicate was repeated 3 times.

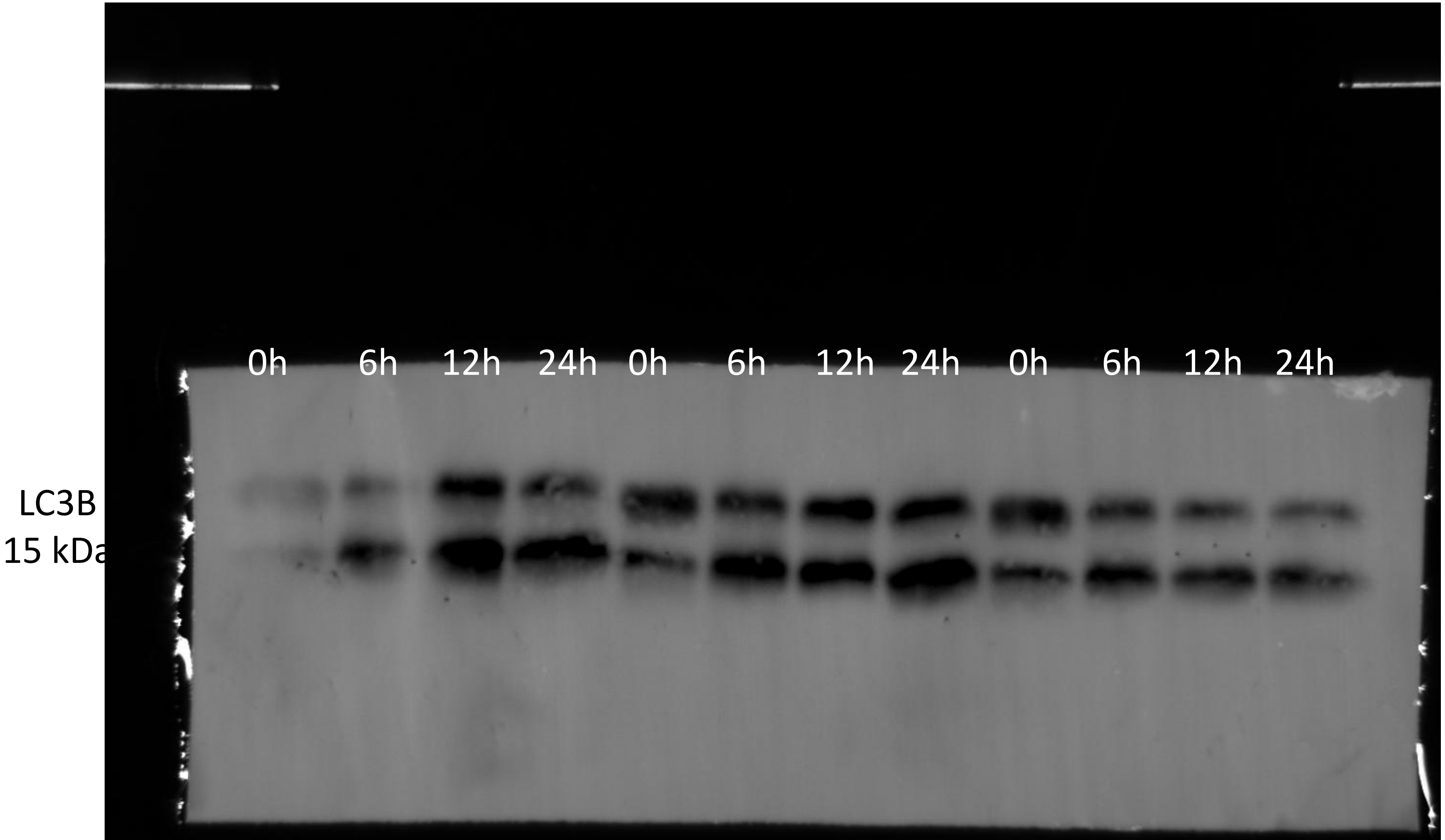

Original image of 4a. The picture is the LC3B of SW480 cell line treated with CAPE for different time. From left to right are 0h, 6h, 12h, 24h. Each replicate was repeated 3 times.

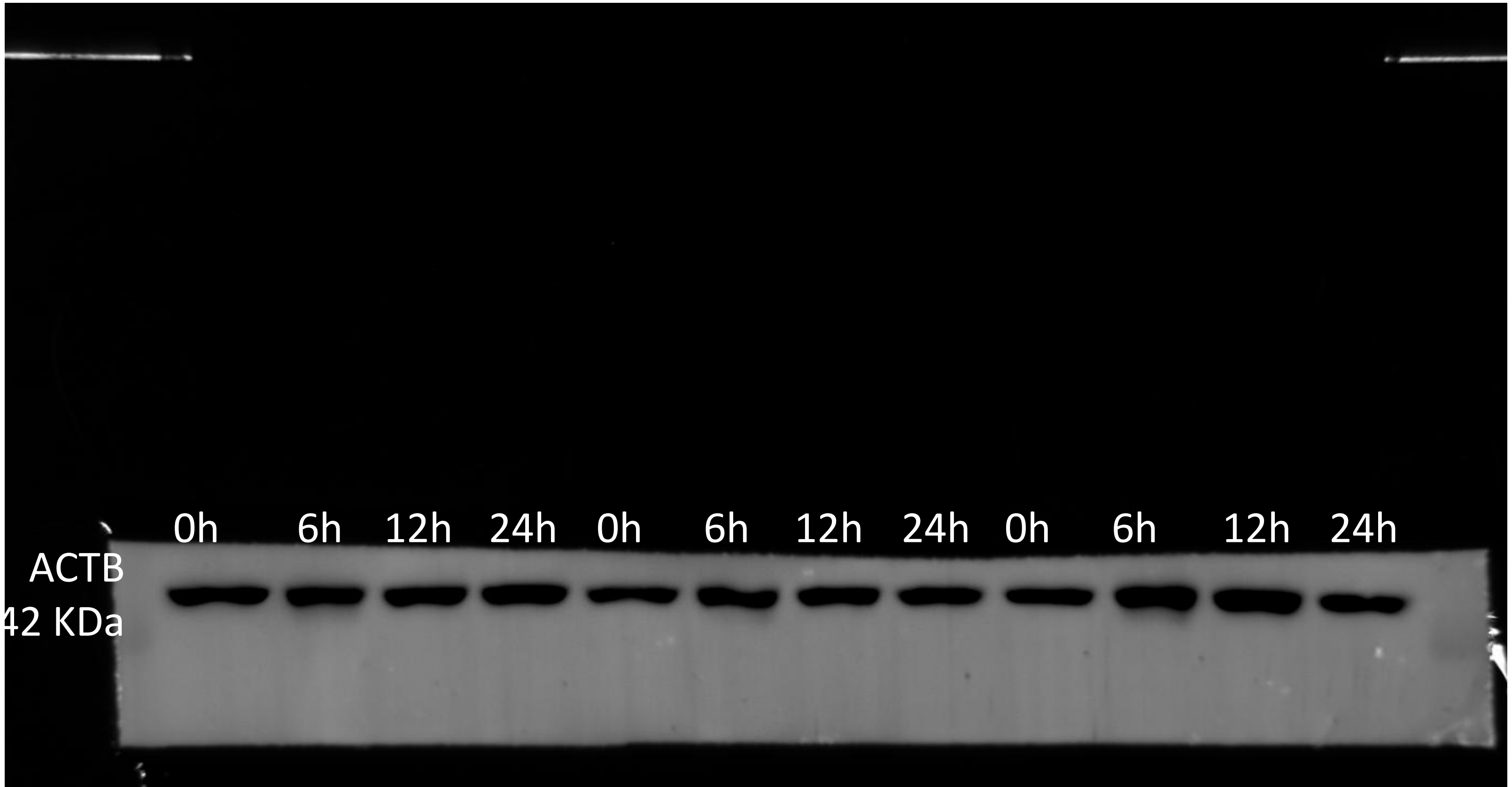

Original image of 4a. The picture is ACTB of sw480 cell line treated with CAPE for different time. From left to right are 0h, 6h, 12h, 24h. Each replicate was repeated 3 times.

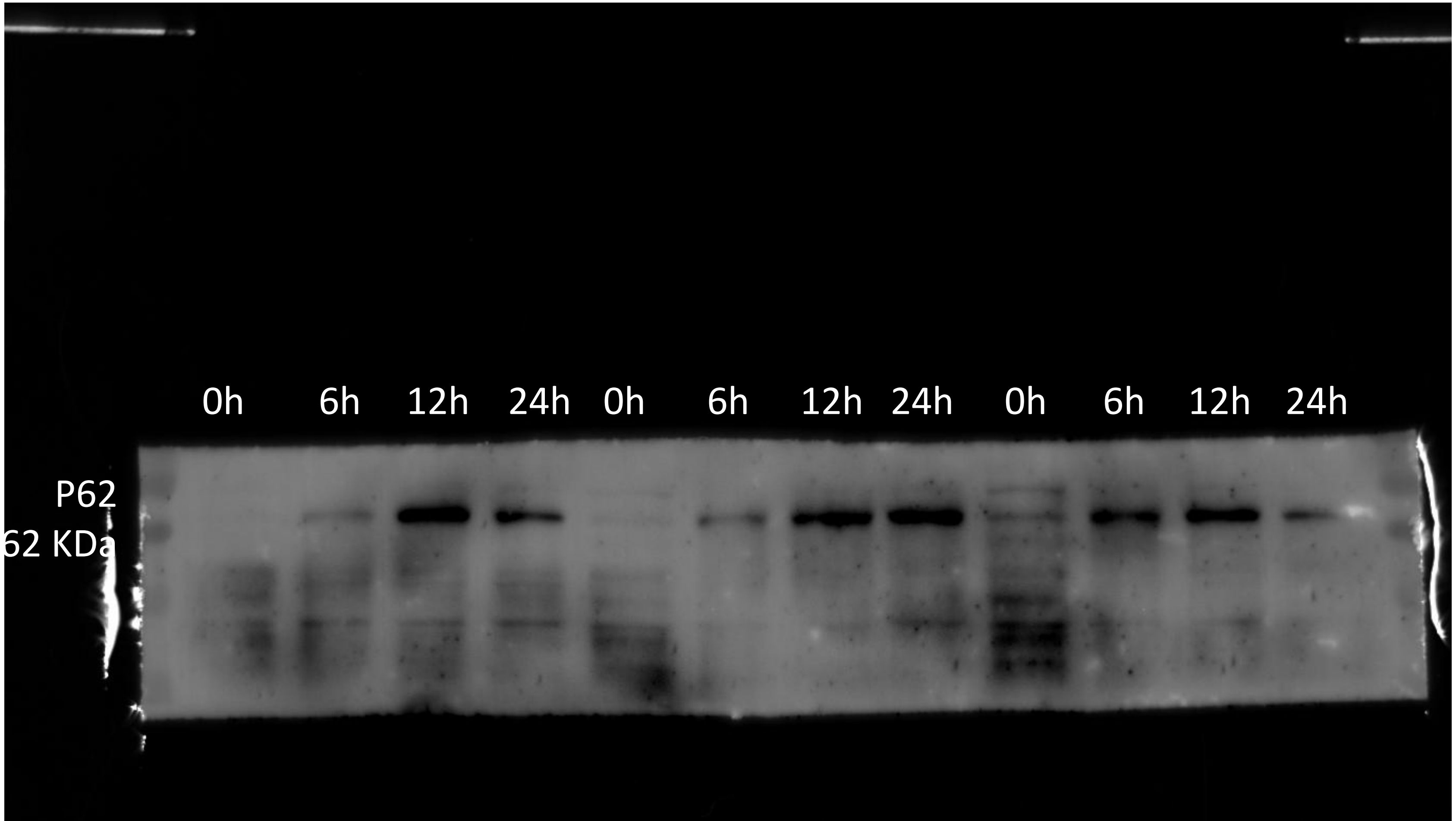

Original image of 4a. The picture is the P62 of HCT116 cell line treated with CAPE for different time. From left to right are 0h, 6h, 12h, 24h. Each replicate was repeated 3 times.

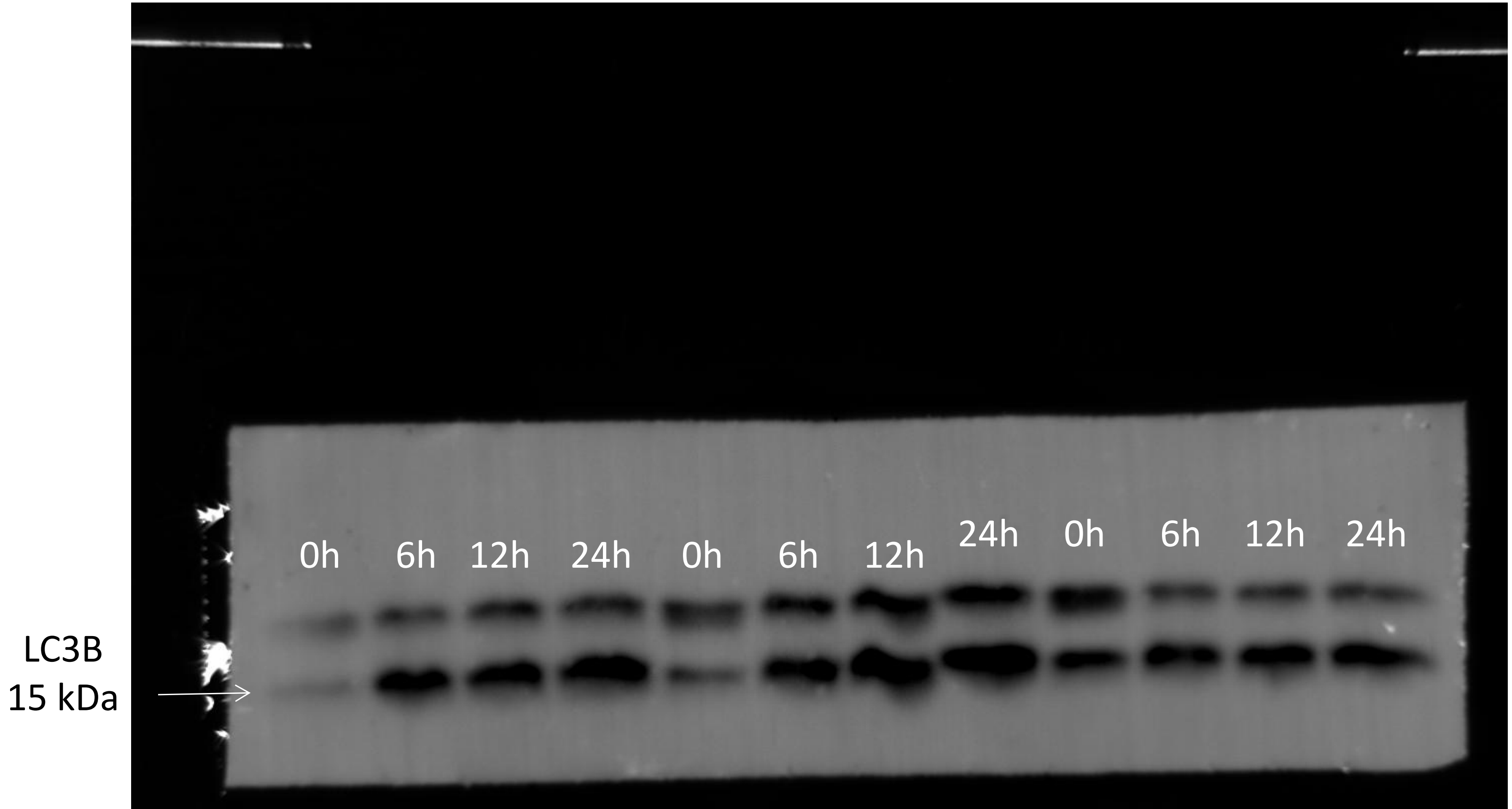

Original image of 4a. The picture is the LC3B of HCT116 cell line treated with CAPE for different time. From left to right are 0h, 6h, 12h, 24h. Each replicate was repeated 3 times.

ACTB
42 KDa

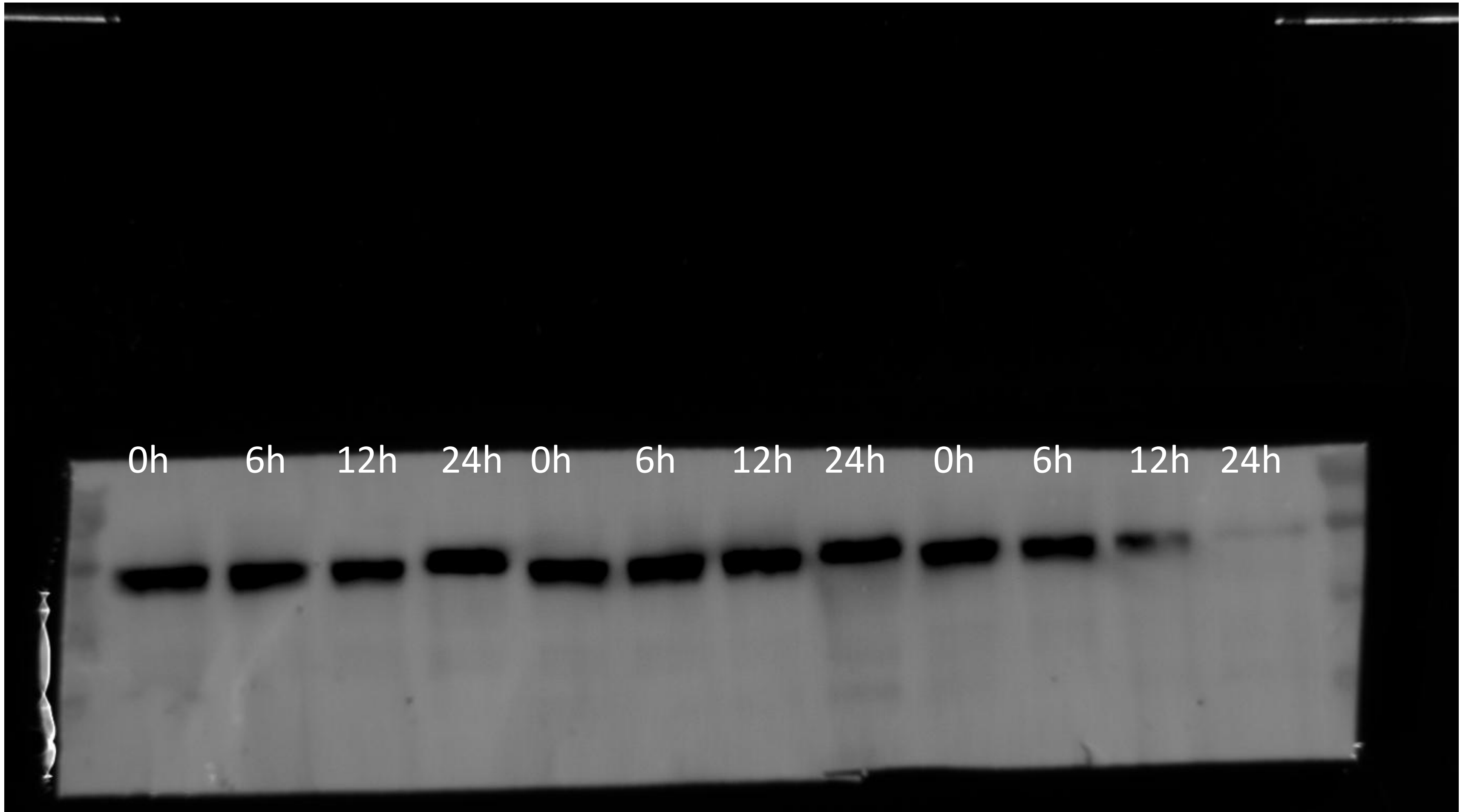

Original image of 4a. The picture is ACTB of HCT116 cell line treated with CAPE for different time. From left to right are 0h, 6h, 12h, 24h. Each replicate was repeated 3 times.

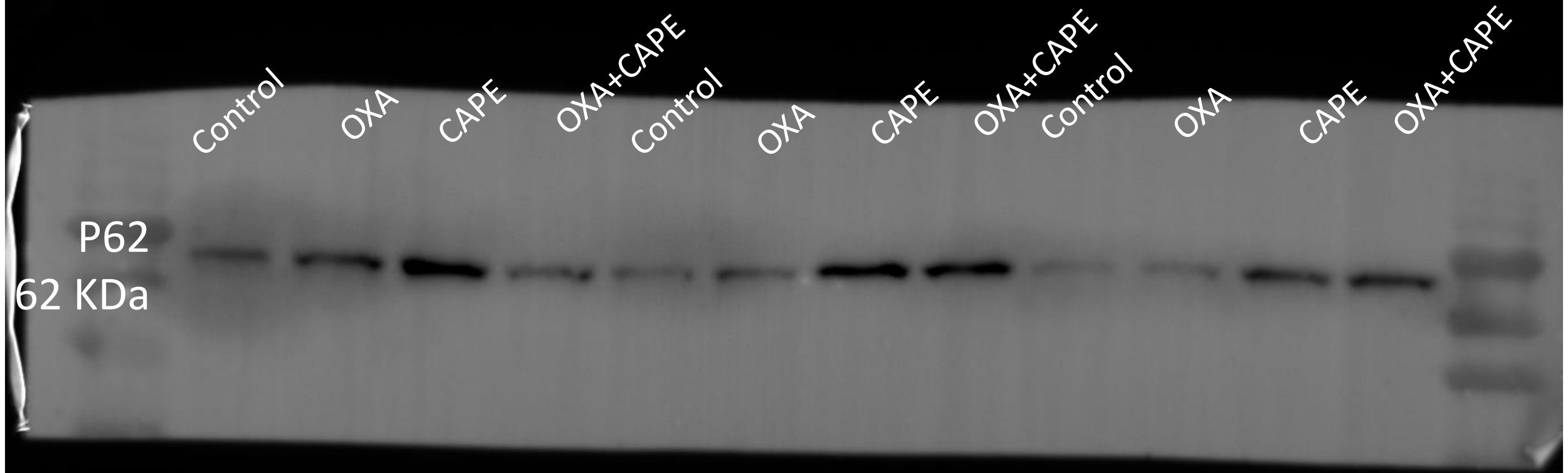

Original image of 4b.The picture is the P62 of sw480 cell line treated with OXA， CAPE， OXA+CAPE. Each replicate was repeated 3 times.

LC3B
15 kDa

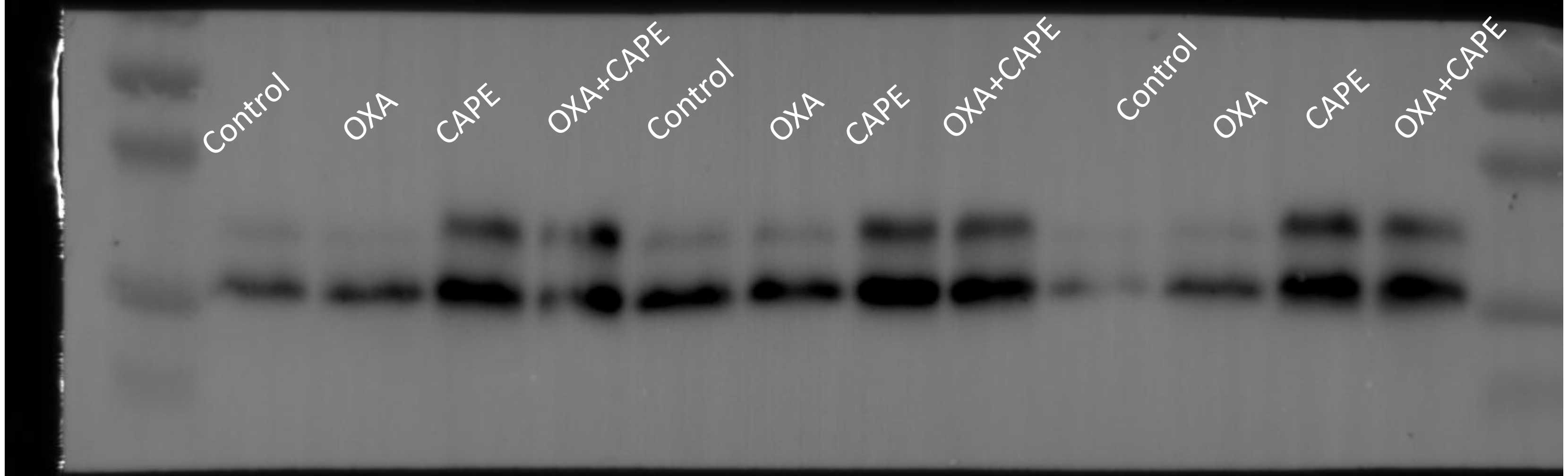

Original image of 4b. The picture is the LC3B of sw480 cell line treated with OXA， CAPE， OXA+CAPE. Each replicate was repeated 3 times.

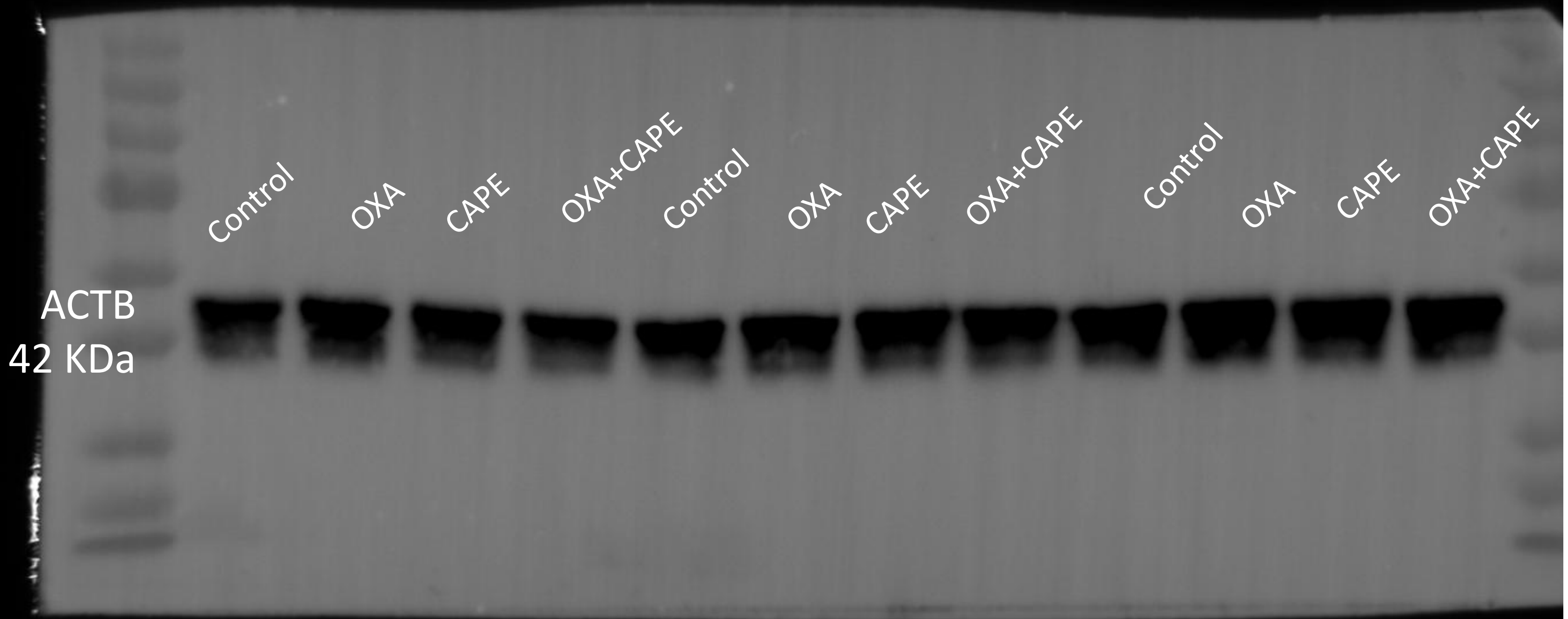

Original image of 4b. The picture is the ACTB of sw480 cell line treated with OXA， CAPE， OXA+CAPE. Each replicate was repeated 3 times.

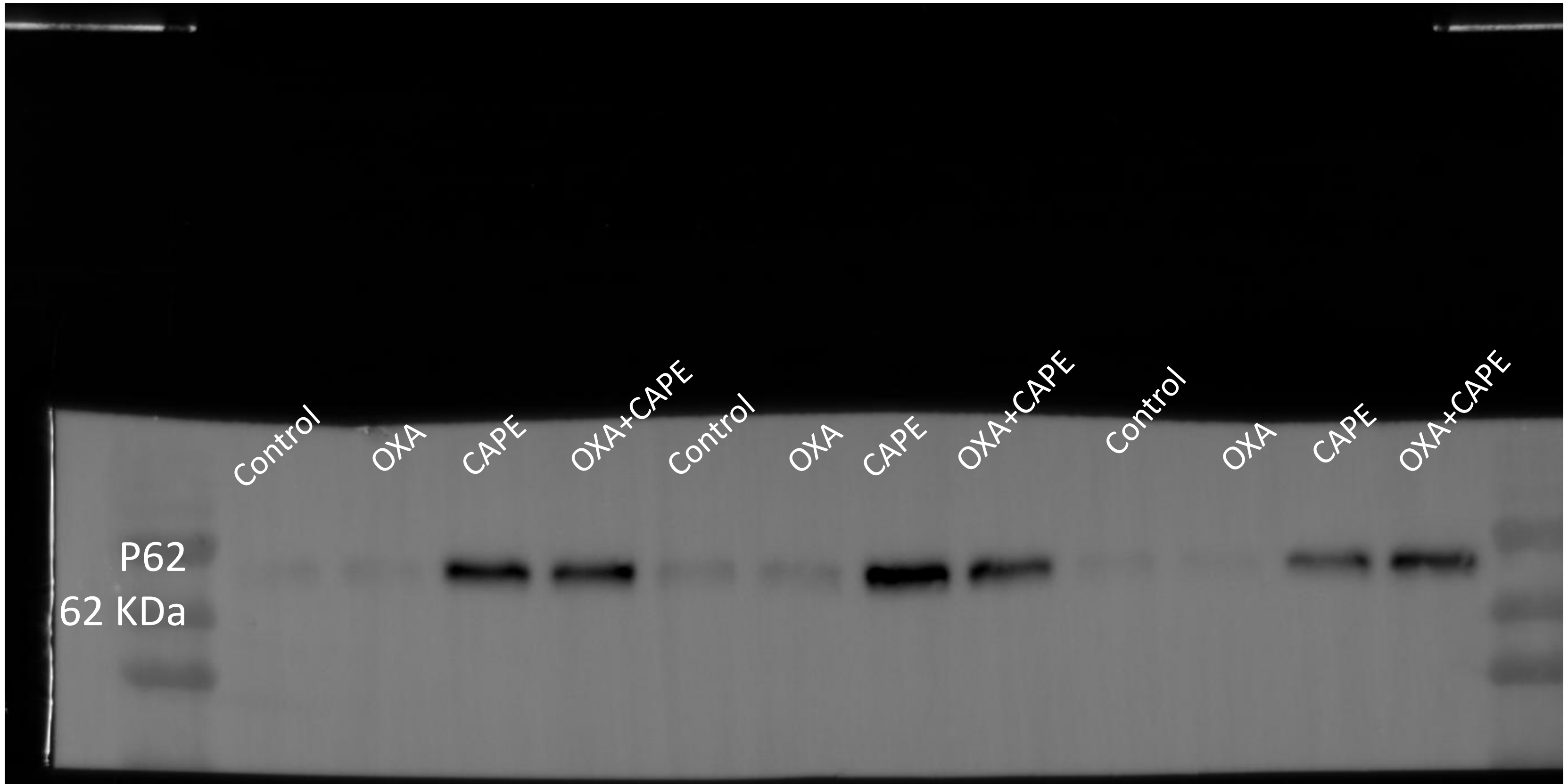

Original image of 4b. The picture is the P62 of HCT116 cell line treated with OXA， CAPE， OXA+CAPE. Each replicate was repeated 3 times.

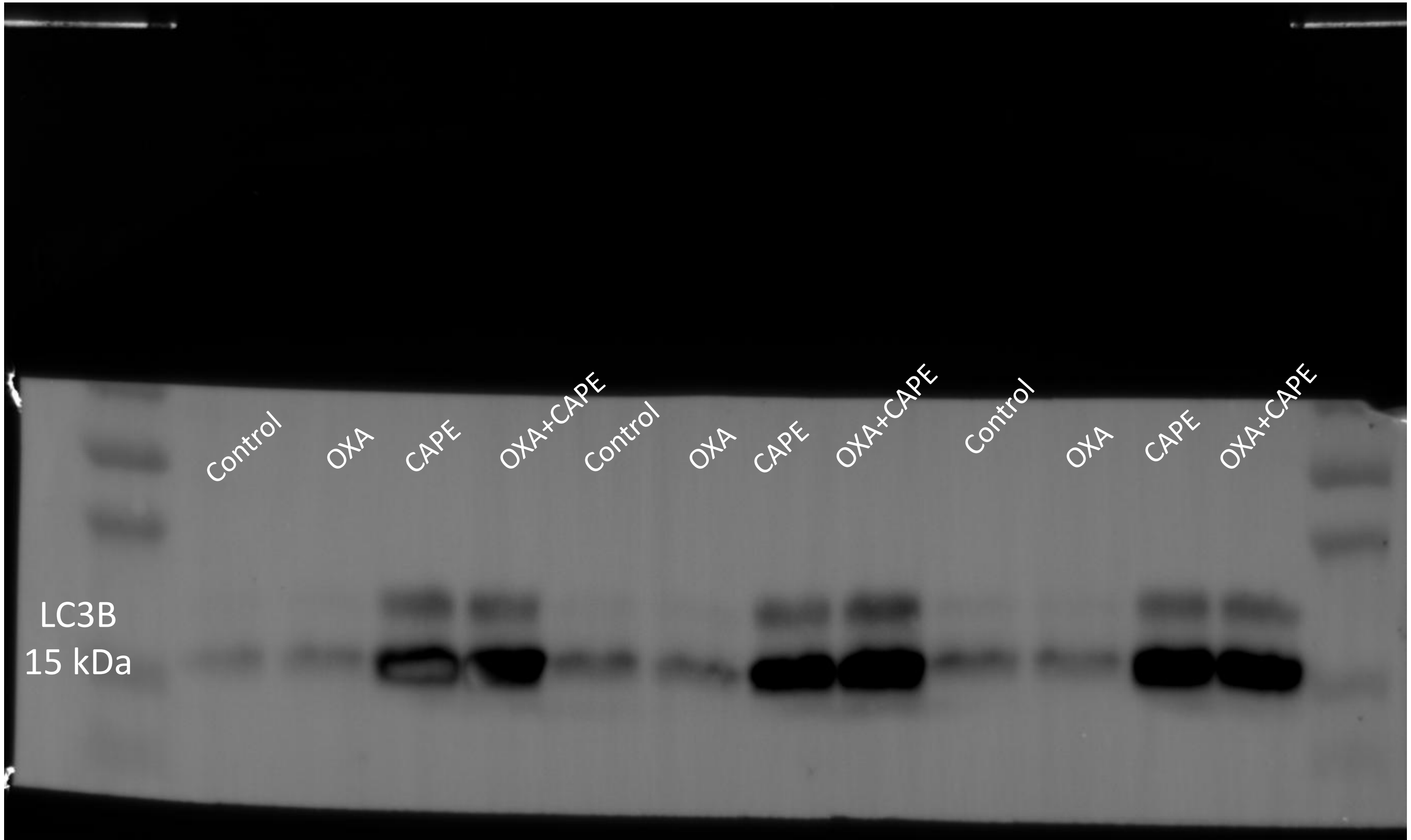

Original image of 4b. The picture is the LC3B of HCT116 cell line treated with OXA， CAPE， OXA+CAPE. Each replicate was repeated 3 times.

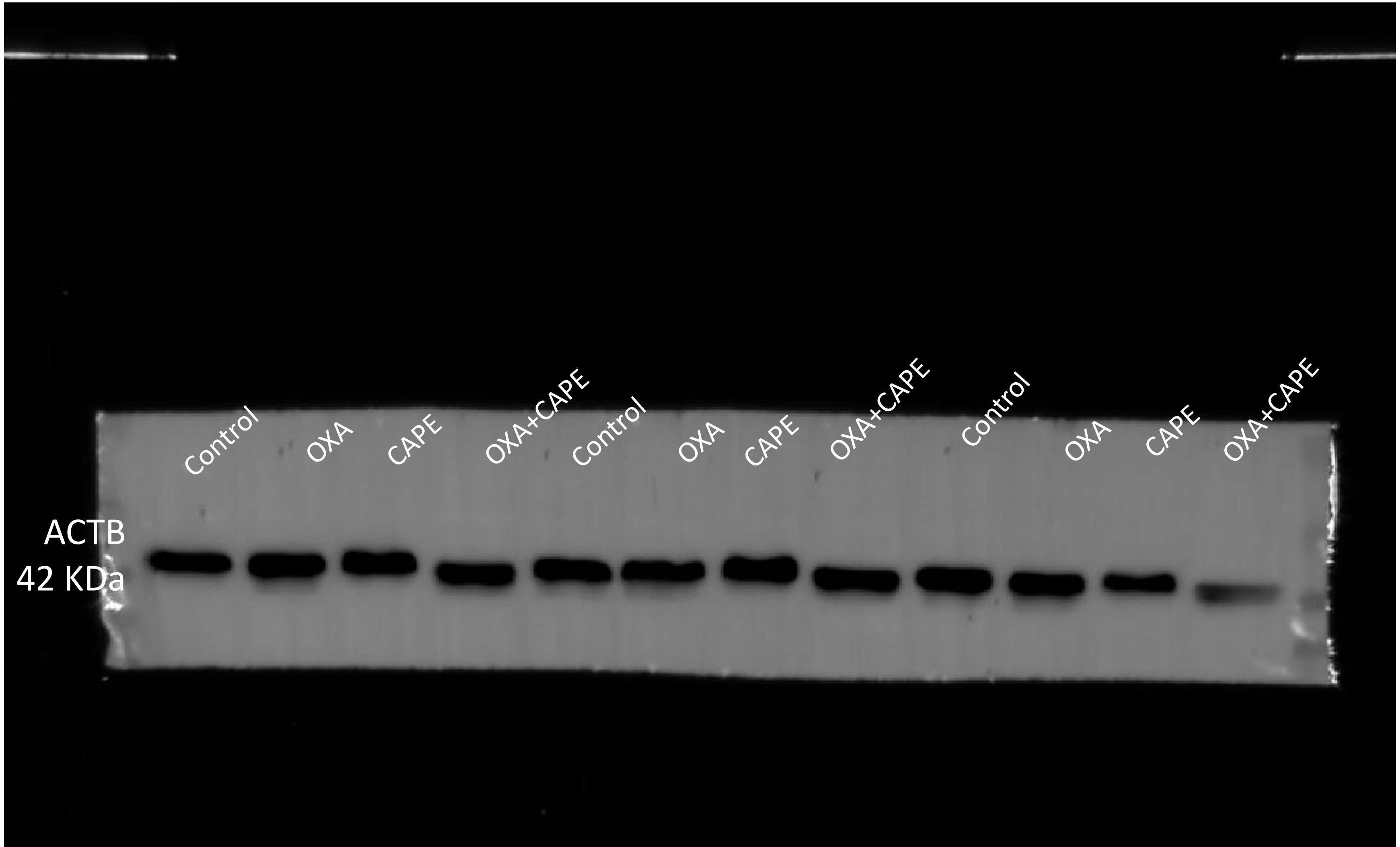

Original image of 4b. The picture is the ACTB of HCT116 cell line treated with OXA， CAPE， OXA+CAPE. Each replicate was repeated 3 times.

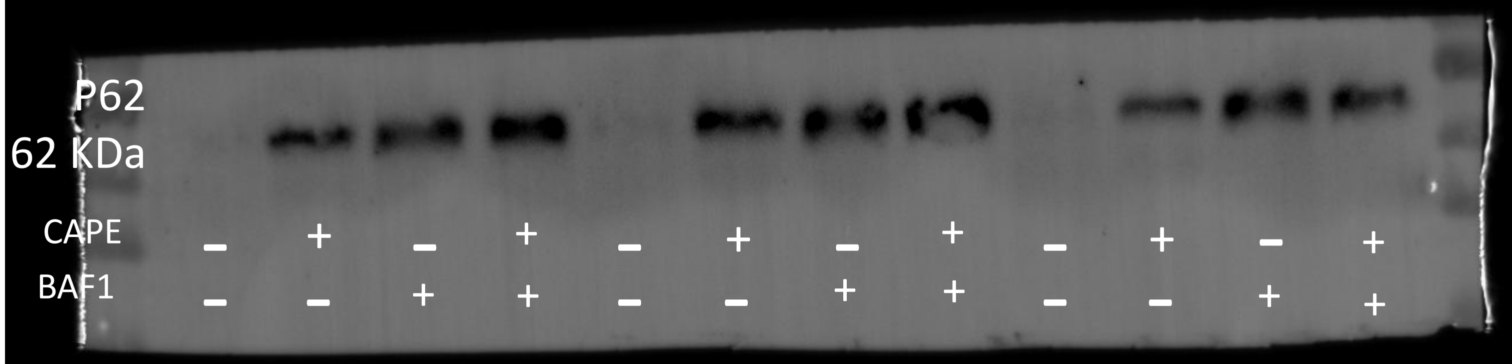

Original image of 5a. The picture is P62 of SW480 cell line treated with CAPE and Bafilomycin A1. From left to right are (CAPE-,BAF-);(CAPE+,BAF-);(CAPE-,BAF+);(CAPE+,BAF+). Each replicate was repeated 3 times.

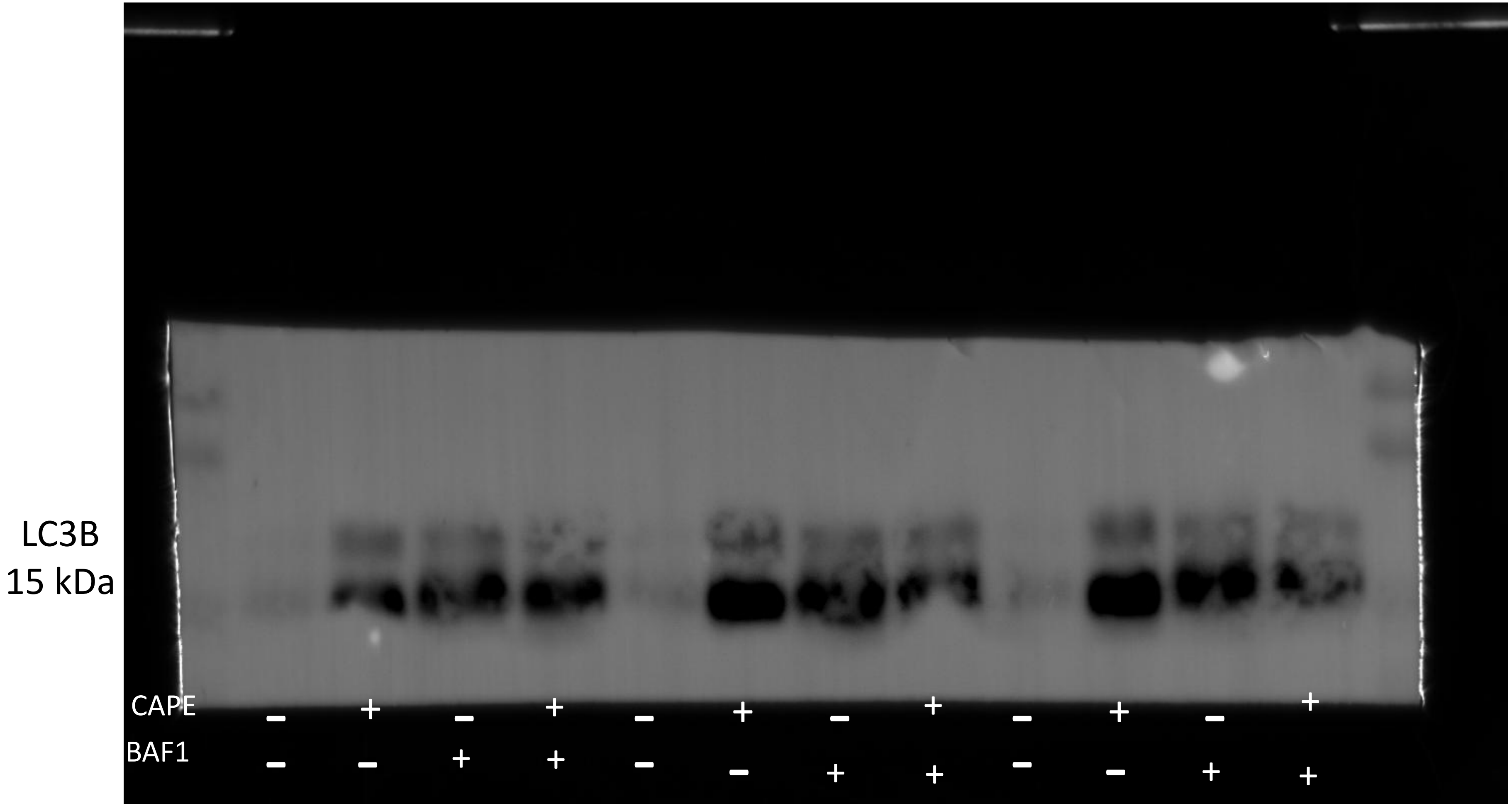

Original image of 5a. The picture is LC3B of SW480 cell line treated with CAPE and Bafilomycin A1. From left to right are (CAPE-,BAF-);(CAPE+,BAF-);(CAPE-,BAF+);(CAPE+,BAF+).Each replicate was repeated 3 times.

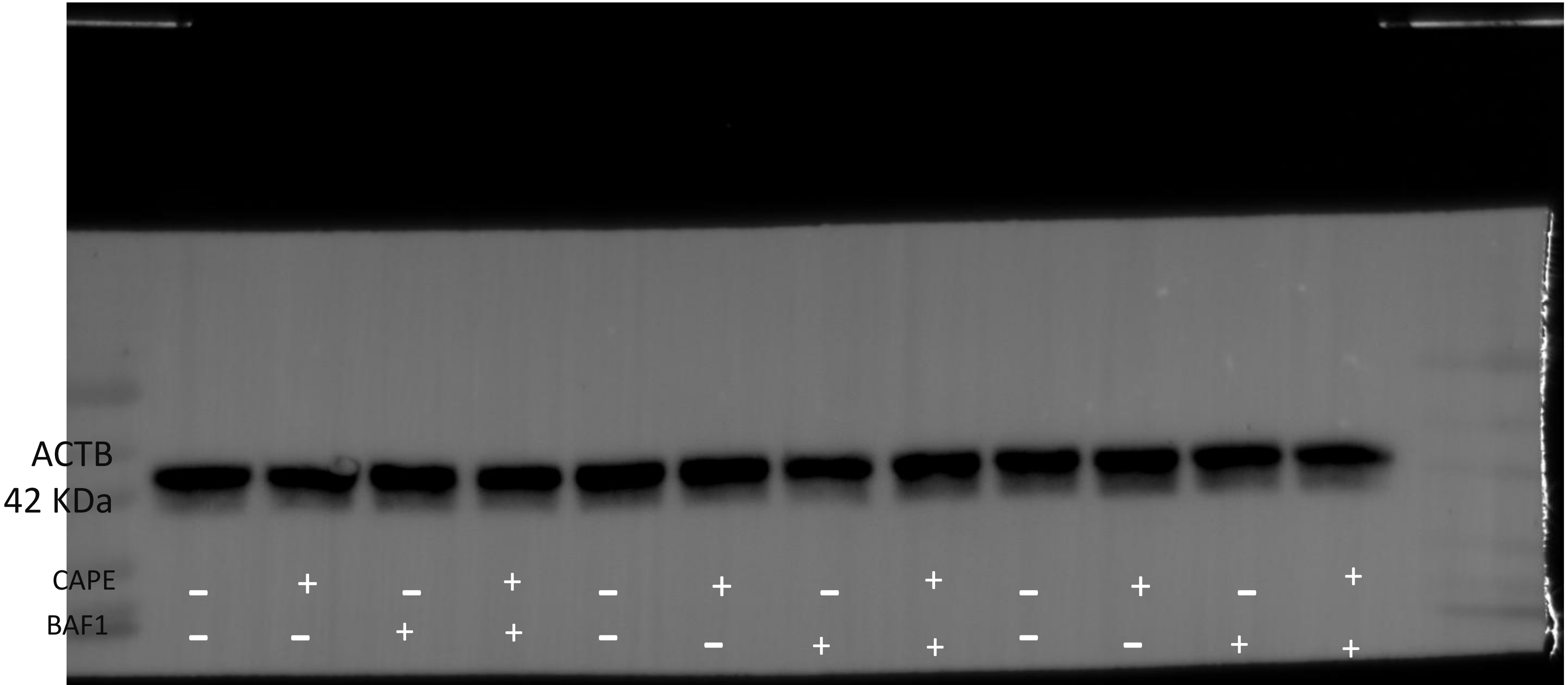

Original image of 5a. The picture is ACTB of SW480 cell line treated with CAPE and Bafilomycin A1. From left to right are (CAPE-,BAF-);(CAPE+,BAF-);(CAPE-,BAF+);(CAPE+,BAF+). Each replicate was repeated 3 times.

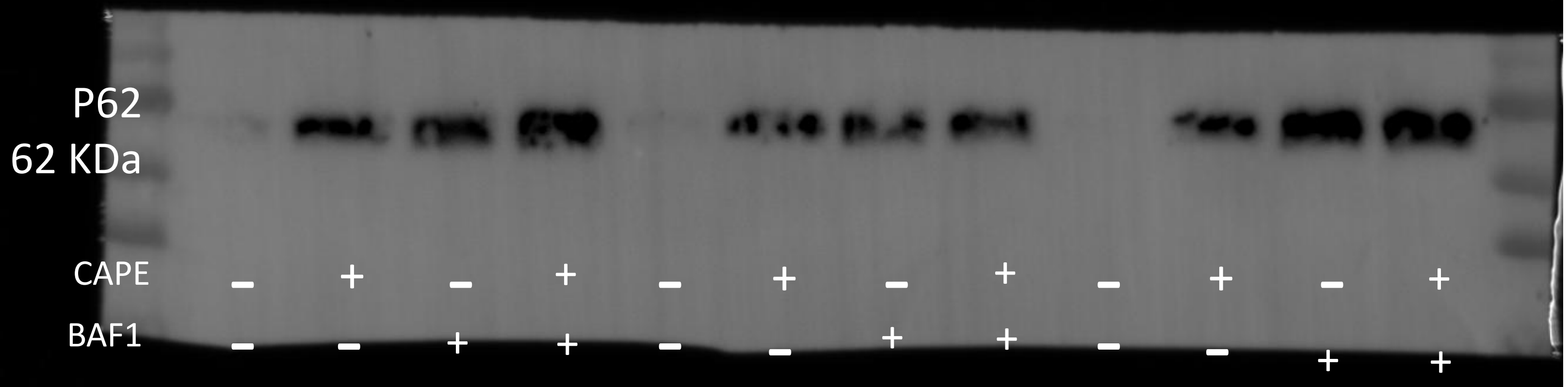

Original image of 5a. The picture is P62 of HCT116 cell line treated with CAPE and Bafilomycin A1. From left to right are (CAPE-,BAF-);(CAPE+,BAF-);(CAPE-,BAF+);(CAPE+,BAF+). Each replicate was repeated 3 times.

LC3B
15 kDa

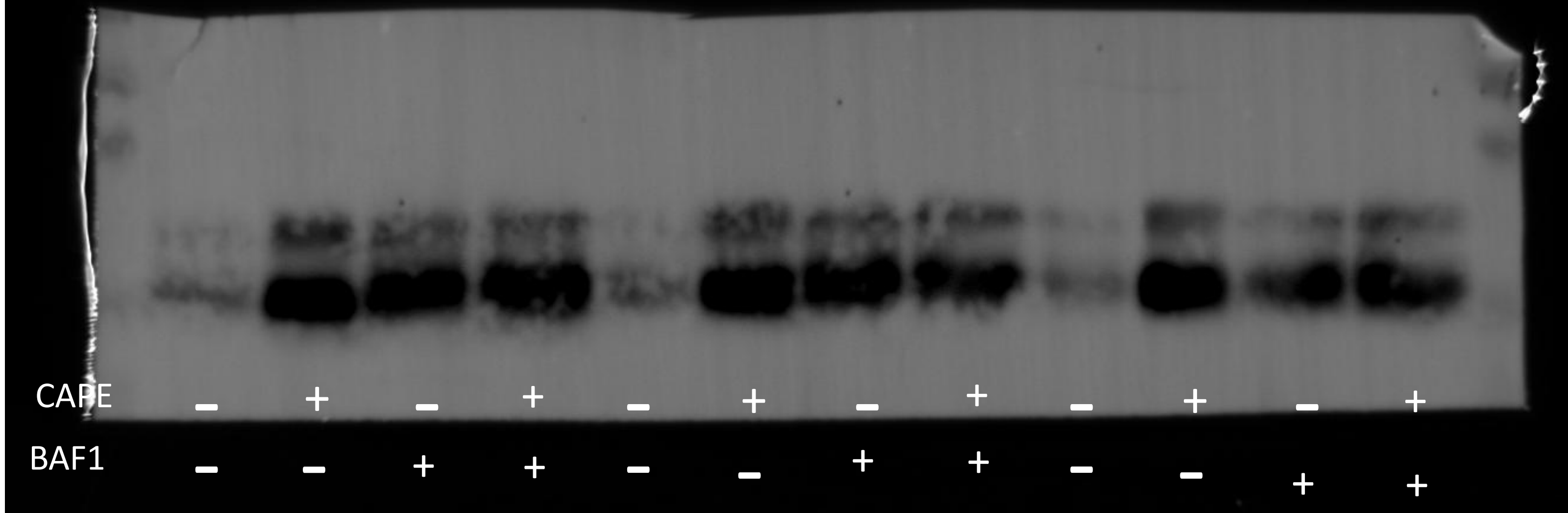

Original image of 5a. The picture is LC3B of HCT116 cell line treated with CAPE and Bafilomycin A1. From left to right are (CAPE-,BAF-);(CAPE+,BAF-);(CAPE-,BAF+);(CAPE+,BAF+). Each replicate was repeated 3 times.

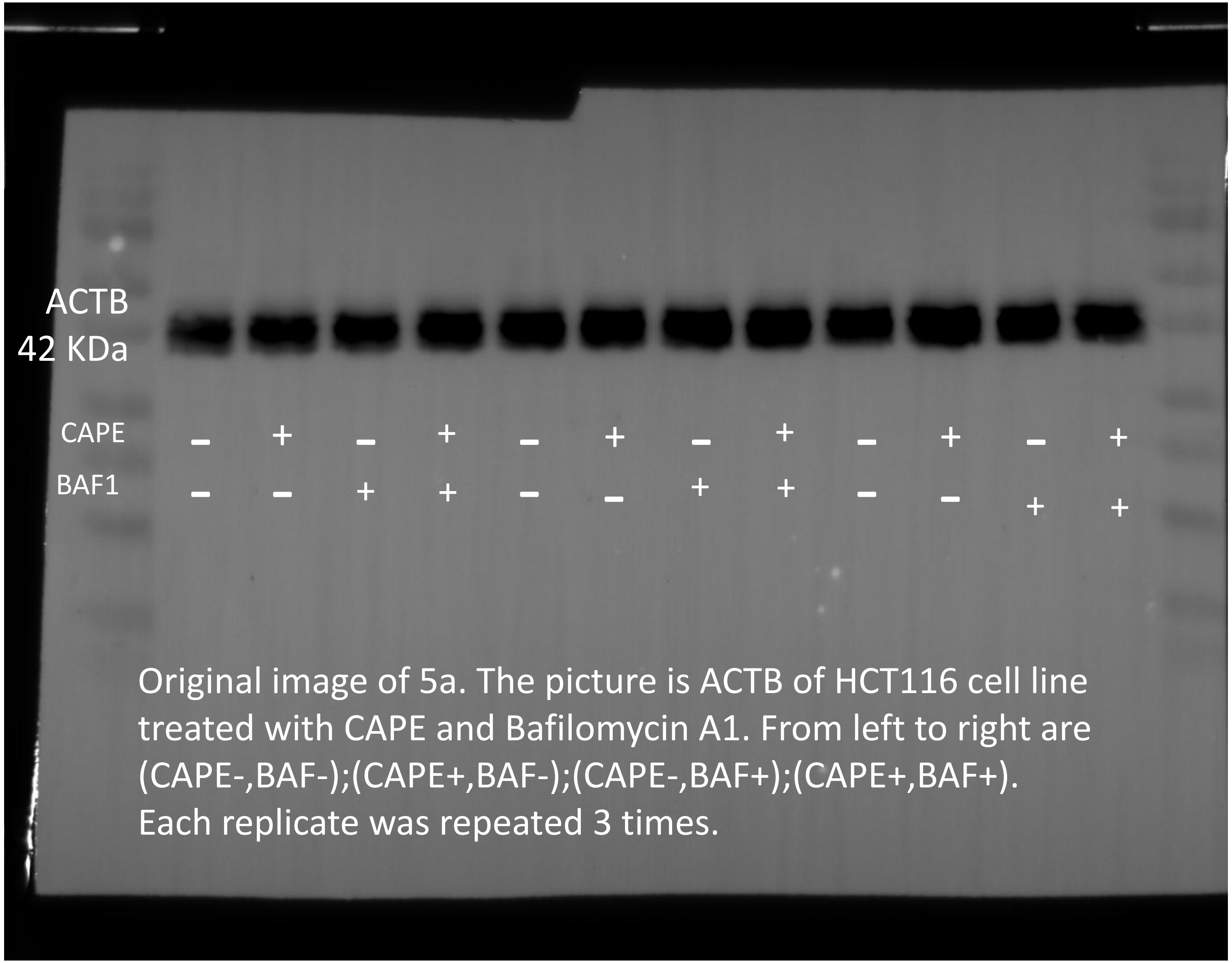

Original image of 5a. The picture is ACTB of HCT116 cell line treated with CAPE and Bafilomycin A1. From left to right are (CAPE-,BAF-);(CAPE+,BAF-);(CAPE-,BAF+);(CAPE+,BAF+). Each replicate was repeated 3 times.
